# Europe’s Rhine power: connections, borders, and flows

**DOI:** 10.1007/s12685-015-0140-z

**Published:** 2015-09-05

**Authors:** Vincent Lagendijk

**Affiliations:** Maastricht University, Maastricht, Netherlands

**Keywords:** Water history, History of technology, Rhine, Transnational history, International organizations

## Abstract

This article explores the pivotal position of the river Rhine in the gradual development of a European electricity system. Although the general image of the Rhine is one of a inland transport corridor, it also acted as a backbone of electricity supply systems since the dawn of the 20th century. By relying on insights from both water history and history of technology, the article argues for a transnational approach to better grasp the dynamics of river use and related electricity generation, which often went below, as well as above and beyond nation-state affairs.

## Introduction

A rather anonymous building in the Swiss town of Laufenburg harbors a control room supervising all international electricity flows in the southern part of the European electricity system. When stepping out of this room, one immediately looks out over the river Rhine and upon the Laufenburg hydroelectric power station. This part of the Rhine was first dammed at the turn of the 20th century and the power plant at the dam supplied energy to both sides of the border formed by the Rhine—Switzerland and Germany. Today this control room, together with another at Brauweiler (near Cologne), controls a European grid that supplies over 500 million people with electricity.

Most people regard the Rhine as a vital transportation infrastructure. Less known, perhaps, is the Rhine’s role as backbone of an expanding energy system. Initially the Rhine’s flow was used to generate hydroelectricity and coal transports, but in later decades also to cool nuclear power plants. This article shows how from small regional beginnings, cooperation on the river’s energy potential grew into a larger international—even European—electricity system. It explores the pivotal role of the Rhine in the construction and operation of the European power grid. Electric power was and is a vital part of Western European economic success.

Historically speaking, the development of the electricity system has been very international, and the Rhine was a major part of that. The central position of Laufenberg speaks to this. This international character is not entirely surprising, as “[r]ivers have a perverse habit of wandering across borders”, in the words of John Waterbury. He added to that that “nation states have the perverse habit of treating whatever portion of them flows within their borders as a national resource at their sovereign disposal” (Waterbury 1979).^1^ While this may be true, cooperation on the Rhine was forthcoming and friendly, with the exception of two phases when respectively France and Germany tried to usurp a large portion of the Rhine energy bounty. Both still had to work through international political bodies, and take into account the physical properties and dispersion of the Rhine’s resources. As the Rhine was reworked, it still was the economic and socio-political context that changed most throughout time.

In uncovering the material underpinnings of the economic ascendancy of this region, this article builds on recent trends within the fields of water history and history of technology. Both in recent years have come under the influence of a transnational historical approach. Doing transnational history implies going beyond national-focused approaches, instead zooming in on actors (people, organizations, rivers) without clear nation-state allegiance, ideas and knowledge, as well as forms of international and sub-national governance.^2^ Transnational historical studies help to expose flows of ideas and knowledge that operate outside national frameworks, without losing sight of the importance of nation-states altogether. Metaphorically speaking, this transnational approach seems best exemplified by rivers, ignoring national borders, influencing societies and economies along the way, while their flow brings matter from one place to another.

By applying such a transnational angle, this article goes beyond more conventional histories of electrification and rivers, which are still primarily national in nature. The article is based on a close reading in history of technology and water history, and incorporates material from national archives (Austria, Switzerland, United Kingdom, and the United States) as well as international organizations (the OEEC, United Nations, and electricity-related ones).

The article continues as following. First, it engages with two strands of literature, by explicating the state-of-the-art in both history of electrification and water history, combined with a review of transnational history. Subsequently, the article will scrutinize the role of the Rhine region in the gradual emergence of a European electricity system. Three specific periods will be addressed. First, a pioneering phase, starting in the last decade of the nineteenth century and lasting until 1914. A second period—a more nationalistic phase—encompasses the Interwar and wartime years. Within this period, ideas for a European network appears on the scene for some years. The third period deals with the post-war years, when European cooperation in the field of electricity was institutionalized.

## Towards a transnational history of electrification and water

In recent years, a real surge in water studies can be noticed. The nexus of water and society is a rich one, fleshing out (wo)men’s wet interactions in a truly interdisciplinary endeavor. Ever-more pressing water needs, leading to struggles and competition between nations and peoples, have sparked historical research into earlier eras.^3^ Arguably the epitome of this trend is the impressive multi-volume series A History of Water, now already in its third series (eight books in total). With its truly global authorship, this series figures many multi-country comparisons and thematic case studies.^4^ Another key volume in this regard is River in History, edited by Thomas Zeller and Christoph Mauch (2008), comparing the development of American and European rivers.^5^ Most studies, however, adhere to a national historical framework when studying the role of water.^6^

Though obviously boosting the nation and national identity, many rivers travers national borders. That, however, should not automatically translate into writing national histories. The French Annales School pioneered problematizing the strict national approach in many regards, combining cultural and social history with economics and geography. Marc Bloch wrote in 1933 that before speaking about the Rhine, one “must first exorcise the demons” (Bloch 1933; Cioc 2004).^7^ Those demons are largely national ones, also according to fellow Annales-historian Lucien Febvre, and the history of the Rhine (also from a long-term perspective) is more than discord and conflict (Febvre and Schöttler 1997). In recent years, several scholars have embraced a non-national and more regionalized perspective. Mark Cioc’s Rhine biography stands out in particular, drawing together the more economic aspects of Rhine development with the ecological history (Cioc 2002, 2004).^8^ More recently, Sarah Pritchard’s Confluence assesses the various forms utilization of the river Rhone through technology. Pritchard shows how this river was important for regional and national identity in France (Pritchard 2011).^9^ In addition, a growing literature on the role of rivers in international development aid and the spread of Western river development schemes assumes a non-national framework (e.g., Biggs 2010; Hoag 2013; Janác 2012; Khagram 2004; Klingensmith 2007; Teisch 2011). Another sub-field of water history zooms in on the legal dimensions of international rivers and the mitigation of international water conflicts (See amongst others Bourne and Wouters 1997; Bruhács 1993; Lowi 1995; Salman 2007; Teclaff 1996; Uitto and Duda 2002; Wolf 1998).

A similar situation exists for the history of electrification. Most historians took a national viewpoint, often telling the tale of electricity’s role in the modernization of the national economy, or the social encounters with a society-changing innovation (see for example Coopersmith 1992; Hallon 2001; Hannah 1979; de Matos et al. 2004; Myllyntaus 1991; Stier 1999; Van der Vleuten 1998). They did so in the footsteps of Thomas Hughes’ 1984 Networks of Power, interpreting electricity networks as so-called large technical systems (LTS) (Hughes 1983, 1987). Such systems should be regarded as socio-technical systems; not only consisting of technological components, but also institutions, natural resources, and legislation. Historians of technology ascribe agency to technological artifacts, similar to the treatment of rivers in several studies as technopolitical entities (White 1995; Pritchard 2011).

There are good reasons for taking a nation-state approach. Nation-states had a significant stake in electrification. As part of official policy, most states aimed to provide the whole country “the means and symbols of modern civilization” (Coutard 2001; Nadau 1994). Technological infrastructures were built to serve specific national socio-economic and political aims. Gabrielle Hecht has labelled this technopolitics, implying “the strategic practice of designing or using technology to constitute, embody or enact political goals” (Hecht 1998). Nation-states thus often promoted electricity systems as stimuli to economic development and to spark modernization, but also to boost national unification. The same holds for national histories of rivers. Here, too, the national state (or lower authorities) tried to “nationalize” this vital wet resource—often related to electricity production (White 1999; Gugerli 1996).^10^

Yet these nationally-focused studies do not treat the international and European dimensions of electricity cooperation to the full extent they deserve. By and large, national-oriented studies seem to downplay the role of non-state actors, cross-border connections, and international institutions—and the interdependencies that came along with.^11^ This cannot be mended through comparing national trajectories only. Therefore this article applies a transnational historical method, to go beyond this “methodological nationalism”.

Coined by political scientists in the 1960s, the term “transnational” was initially used to depict forms of interaction between non-state actors across national boundaries (Patel 2004). More in general, transnational history investigates the circulation and flows of people, ideas and objects across national boundaries, as well structures facilitating these flows (Saunier 2008). In recent decades, the work of international organizations also became an prominent object of study for transnational historians.^12^ Both rivers and technologies fit such an approach. In recent years, historians of technology picked up this approach to analyze European integration. In a pioneering article, Misa and Schot labelled the technological foundations as a form of “hidden integration”, as it was previously missed by European historians (Misa and Schot 2005; Kaiser and Schot 2014).^13^ In processes of a network-building, designing “Europe” has not always been the aim, but sometimes emerges as the outcome of attempts to manage “cross-border flows of people, goods, information, or energy” (Schipper and Schot 2011).

This brings us to the Rhine region. Rhine cooperation is often assumed to be a precursor to European integration, and although there are certainly several synergies, there is little systematic evidence for this (Scott 1989; Knippenberg 2004). Still, it clearly was the cradle for many industrial firms, boosting German-Dutch economic growth, but also a space where new institutions like the Central Commission for the Navigation of the Rhine (CCNR 1815) was born (Henrich-Franke and Tölle 2011). From an infrastructure perspective, the Rhine region was a prime focus (and destination) for Dutch, Belgian, and of course German railway companies (Schot et al. 2011). The Rhine itself also was the subject of ambitious river transport schemes, as linkages with the Rhone and the Danube were envisioned already in nineteenth century.^14^

Similar—technological and institutional—trends can be identified for the region in the field of electricity. Although the electrification of the Rhine region was not the solely determining factor leading to a European system, it definitely played a pivotal role in the process towards it. The subsequent sections will discuss these flows within and beyond the Rhine region, showing how the intertwinement of the Rhine electricity systems sparked the emergence of a European grid.

## The pioneering phase

With a length of 1300 km the Rhine is the third longest European river, behind the Danube and Volga. It serves a basin of 170,000 km^2^ throughout seven countries: Switzerland, Austria, Germany, France, Luxembourg, Belgium and The Netherlands. More than 50 million people live in this basin today (Huisman et al. 2000; Wieriks and Schulte-Wülwer-Leidig 1997). By the start of the twentieth century, the Rhine’s wild nature had been tamed, leaving its once plentiful salmon almost extinct. The Tulla Rectification Project (1817–1876) played an important role in this transformation. Named after its architect, Johann Gottfried Tulla, this project in the first place tried to curtail the river’s flooding. This implied shortening the river by some 82 km and standardizing its width between 230 and 250 meters between Basel and Strasbourg. This vast riverine project brought various unintended consequences, particularly for the riverscape’s nature. Harm was done to the breeding ground of fish, and diminished the bird population. The ecosystem of the river was further degraded by pollution from industry settling on the Rhine’s banks (Cioc 2002; Tümmers 1999).

Industries were drawn to the Rhine basin’s natural resources, which included vast coal resources. During the late nineteenth and early twentieth century, the Middle Rhine saw most action; new canals were dug, railroad tracks were laid, and port facilities were built in order to exploit these resources. Particularly the Ruhr area, named after the tributary stream of the Rhine, became synonymous with coal and iron (Chandler 1990; Cioc 2002). The chemical industry followed in the slipstream of mining and metallurgical firms, leading to ever-increasing water and energy demands.

Around the turn of the century, the High Rhine took the first steps towards electrification. This was an international affair from the start, as electricity was transmitted across borders. At least three reasons help to explain this. First, the High Rhine formed the political boundary between Switzerland and Germany. Constructing a dam with a power plant at Rheinfelden in 1894 thus required a bilateral agreement between the Swiss canton Aargau and the Grand-duchy of Baden (Kleisl 2001). After Rheinfelden, ten more dams—including the one at Laufenburg—became operational on the High Rhine between 1912 and 1963.^15^

Growing entrepreneurial and industrial activities provided further impetus for cross-border cooperation, and an increasing demand for energy. The case of Rheinfelden power plant illustrates this, constructed under supervision of Emil Rathenau, chairman of Algemeine Elektrizitäts–Gesellschaft (AEG) (Cioc 2002). Rathenau did not just finance the power station, but also an aluminum and chemical plant which both constructed plants near the dam, and became large electricity consumers. Other energy-intensive chemical and aluminum industries followed, and settled on both the German and Swiss banks of the Rhine (Rathenau 1985). By attracting these large consumers Rathenau ensured a good load factor for the Rheinfelden plant. Load factor is the ratio of the average load to the maximum load. In short, the “flatter” the curve, the better the load factor. Ensuring a sufficient consumer base is key as storing electricity is very difficult; hence, supply and demand have to be balanced. The challenge for power station managers is finding a customer base leading to a constant load, even if this means meeting energy demands across national border.

Combining High Rhine hydropower with other types of electricity production are a third reason for international cooperation. Each type of electricity generation has its own pros and cons. While coal-fired plants provide a stable production, this comes at relatively high and fluctuating fuel costs. While hydropower depends on the seasonal variable availability of water and requires substantial initial investments, this comes with little running costs. Balancing between various resources—a good economic mix—is thus profitable. This was possible for Upper Rhine power plants as adjacent regions offered complementary resources. Switzerland harbored more Alpine hydropower than it could consume, while Germany owned considerable coal resources. Neighbors Austria and France, or even more distant Poland, had hydro or coal resources, and cooperation with them also offered potential benefits. Technological innovations during this period, as the introduction of 110 and later 220 kV transmission lines, enabled material connections between the Upper Rhine and these more distant resources.^16^

But these power plants and transmission networks required large investments. In order to obtain sufficient funds, relations between the electrical industry, electricity utilities and financial institutions became close. Manufacturers of electrical equipment joined forces with banks to form multinational holding companies of a type called Unternehmersgeschäft, in order to raise the capital required for larger power plants and high voltage transmission lines (Chandler 1990; Hausman et al. 2008). Rathenau and Algemeine Elektrizitäts–Gesellschaft (AEG 1887) for example incorporated the Bank für elektrische Unternehmungen (Elektrobank), supported by Swiss and German banks.

Elektrobank and others were crucial in expanding the local networks to wider scale around the Rhine. The system of the Rheinisch–Westfälischen Elektrizitätswerks Aktiengesellschaft (RWE 1898) probably offers the best example. RWE made good use of the diversity of fuel types along the Rhine and neighboring regions, resulting in a well-balanced economic mix. The extension of the RWE grid to the south, tapping into the Rhine, Neckar and Alpine regions in Germany and Austria, was financed by Elektrobank (Boll 1969; Hughes 1983). The cheapest suppliers of electricity—brown coal plants at the Ruhr mine heads—provided the year-round base load, while hydroelectric plants with water storage capacities—like the Vermuntwerk—covered seasonal shortages and peak loads. A switching station was installed in Brauweiler in 1929 to facilitate optimal operation of the system.^17^ The Brauweiler station today monitors the northern part of the European system, like the Laufenburg station does for the south.

In the first two decades of twentieth century no explicit reference was made to a future European system, nor were concrete plans for national systems formulated. The transmission lines connecting hydro with coal-fired plants in the Rhine area came about through private initiative, with little involvement of political authorities. Mining, metallurgical and chemical industries thereby strengthened the cluster emerging around the Rhine.

## The nationalist phase

This changed during and after the First World War, as nationalistic tendencies gained strength, while at the same time European ideas were voiced for the first time. In the 1910s and 1920s, national authorities increased their grip on the electricity sector via new forms of legislation, increasingly hampering the construction of new cross-border lines and international electricity flows (Hausman et al. 2008). Authorities shifted the focal point towards constructing transmission lines within their respective borders, thereby containing energy resources for benefit of the national economy. Partially in response to these nationalistic tendencies, engineers started to suggest schemes for a European electricity system towards the end of the 1920s.

Most states along the Rhine displayed protectionist and nationalist reflexes, where debates focused on nationalizing hydropower resources (Gugerli 1996; Varaschin 1997). International political issues, resultant from the war’s aftermath, hampered cross-border electricity cooperation around the Rhine. Not only did the Versailles Treaty return the regions Alsace and Lorraine to France; France also received the single right to exploit the Upper Rhine—now the border with Germany—for irrigation and electricity generation. This allowed France to withdraw all the water required for electricity generation, granted that Germany contributed to the necessary costs, and that France repaid Germany half of the value of electricity produced.^18^

Having the upper hand on the Rhine, France embarked on a plan for a parallel canal on the Upper Rhine between Strasbourg and Basel, the Grand Canal d’Alsace—thereby circumventing the need to compensate Germany. What had surely helped, was French support for extending CCNR membership with allies like Great Britain and Belgium, and the French right to appoint the president (Disco 2006). The proposed Canal would connect the (now) French Alsace cities of Colmar, Mulhouse with Basel and Strasbourg, originally included eight hydropower plants and shipping locks, and hoping to rejuvenate the slumping French economy (Cioc 2002; Moser 2004; Tümmers 1999). The plan drew strong criticisms from Germany and Switzerland. Swiss power companies feared French competition in electricity production, and several Rhine harbor cities in both countries feared the adverse effects of water withdrawals on Rhine river transport. After considerable French concessions in 1922, preparations for the hydropower plant at Kembs did start. This plant eventually was the only one of the Canal, and co-financed by Elektrobank (Disco 2010).

The political climate of the Rhine region remained nevertheless tense. German and Allied governments quarreled over German war reparation obligations, eventually leading the French to occupy the Ruhr area in early 1923. French and Belgian troops seized timber, cokes and coal in order to force the German government to continue to pay reparations. Yet even after the occupation spread to parts of the Rhineland, and the French planned for an autonomous Rhineland state, the Germans refused to budge. The 1924 Dawes Plan broke the deadlock by restructuring German reparations, effectively ending the occupation of the Ruhr (Steiner 2005). The Locarno Treaty, signed a year later, opened up a period of relative calm political waters by guaranteeing the Franco-German border and rehabilitating Germany’s political stature.

This period of relative optimism brought new opportunities for international technical cooperation. In 1926 engineers from Germany, France and Switzerland presented a plan to generate hydropower and to exchange electricity along the Rhine and beyond (Wiedenmuller 1926). Again these proposals proposed to combine complementary resources (economic mix) across borders in the Rhine region. Some went even further. In 1929 and 1930 at least three schemes for a European network were presented at international engineering venues, and would prove to have lasting effects (Schönholzer 1930; Viel 1931). The best-known of these was by German engineer Oskar Oliven, director of the Berlin-based Gesellschaft für Elektrische Unternehmungen (Oliven 1930; Lagendijk 2010).

These ambitious, if not utopian schemes merged both technical–economic principles with ideological and techno-political aims. The schemes proposed European-wide electricity networks connecting a wide range of complementary resources ranging from Norwegian hydropower to Silesian coal-fired plants, providing for a good economic mix, and linking all major European urban and industrial centers as well. The construction of such an extensive grid would also stimulate economic recovery, requiring a lot of labor and capital investments. In terms of ideology, these engineers perceived infrastructures—like electricity networks—as forceful techno-political instruments. A European electricity network would, it was thought, physically tie European nations together and thus create strong mutual dependencies.^19^

The League of Nations and the International Labor Organization embraced schemes such as Oliven’s in the context of their plans for European unification. Set in motion by French foreign minister Aristide Briand, and supported by the European movement and by the ILO-director Albert Thomas, the LoN studied several possibilities of further European cooperation (Fleury and Jílek 1998). This included a European electricity grid, which was under discussion from 1929 until 1934. The notion of a European system gained prominence in engineering circles and beyond, to little avail, but lingered on after 1945.

After this European intermezzo, the Rhine re-emerged as a border between two antagonizing states. States again prioritized national interests during the 1930s. In 1938, the French government announced a national interconnection program, expanding the French network with 4185 km costing 1.5 billion French Francs. One-third of this was earmarked for transmission lines in the interest of national defense, primarily located in the north-east of France, where power plants were vulnerable in case of a German attack (Bouneau 1994). This new network supplied the ill-fated Maginot Line with electricity, for provisioning trains, ventilation systems, and electrified gun turrets (Morsel 1994). A central dispatching bureau was also part of the national program, coordinating information exchange between regional dispatch offices, helping to improve the economic mix between thermal and hydroelectric power. In addition, in case of a war such dispatch bureau could allocate electricity to key sectors and areas (Bouneau 1994).

On the eastern side of the Rhine the importance of the electricity system in case of war was equally recognized. Like in France, a national German load dispatcher was installed for both economic and military reasons (Stier 2006). The Ministry of Aviation (Reichsluftfahrtministerium) tested the possible damage of possible air raids and artillery attacks by tying explosive charges to transmission lines. Strong increasing Germany electricity needs presented a more structural problem. The energy-intensive economic policy almost “forced” the National Socialist regime to search for additional electricity sources in occupied countries and annexed regions. Nazi planners in particular targeted Austria, and after the Anschluss in 1939 work started on hydroelectricity plants on the Inn, Enns, and Donau. Alpine storage plants like the Tauern–Großkraftwerk at Kaprun were envisioned to cover peak loads (Maier 1993). Nazi energy hunger boosted network-building in Europe, with ambitions eventually reaching beyond Austria. In 1941 the Nazis discussed building new connections from Austria to Bulgaria, Slovakia, and Croatia, and the occupation of Norway in April 1940 revived ideas of importing Norwegian hydroelectricity.^20^ By the end of the war little had materialized, except for a connection between the dispatch center in Brauweiler with the Netherlands and Belgium. This proved to be an important link after the war (De Heem 1952; Verbong et al. 1998). Links with neutral Switzerland were also reinforced during the war years. Swiss electricity exports reoriented towards Germany, from 25 to 58 % of total electricity exports. This included Swiss exports to occupied France, but exports also rose because of several new plants on the High Rhine, like Ryburg–Schwörstadt and the Aarewerke (Kleisl 2001).

Private actors, too, continued to make their mark on international electricity relations. In several cases, non-state actors continued to represent their interests during wartime. Discussions on new German–Dutch connections took place under supervision of the responsible Dutch ministry, but were conducted between managers from and representatives of two Dutch provincial electricity companies and RWE (Verbong et al. 1998). In Switzerland private actors remained influential, too. A new clearing agreement between Switzerland and Germany—favoring the latter—came about through pressure from banks such as Crédit Suisse—which had made huge investments in electricity—and powerful frontier power plants such as Laufenburg and Ryburg–Schwörstadt, who sought to ensure and stabilize their income (Kleisl 2001).

Historian Bernard Stier has argued that the Nazi period provided fertile ground for the construction of a European system. Although the ideological inspiration differed remarkably from the European intermezzo in the 1930s, he has a point in terms of network-building. Connections between Germany and its neighboring countries were all strengthened between 1939 and 1945 (Stier 2006). Paradoxically, the German system became more “European” at the height of the nationalist phase.

## The Rhine as an axis in institutionalized European cooperation

While the wartime brought better interconnections, European cooperation received a further boost after 1945 when electricity became a priority for planners. Whereas before the war authorities were primarily interested in steering developments within their national borders, they now institutionalized international collaboration. The Rhine region, at the time the best interconnected area within Europe, was the central axis in this process. But before fruitful mechanisms could be founded, the future of the Rhineland had to be settled first.

Shortly after Germany’s surrender, the United States Supreme Headquarters Allied Expeditionary Force in Western Europe organized a meeting in Laufenburg, Switzerland. The participating states (Switzerland, France, Belgium, the Netherlands, and the Allied occupied zones of Germany) discussed how to make better use of available electricity, with several regions suffering under electricity shortages. It was agreed that parts of Belgium, France and the Netherlands received portions of the production of power plants on the Rhine. This went largely unused with Germany’s economy in ruins.^21^ Newly constructed transmission lines between the German-Swiss Rhine plants and Kembs helped solve post-war shortages in neighboring countries.

Negotiations on reconstruction were sometimes shot through with tension. One example is the dispute over electricity from western Austria. Technical circumstances—the Austrian network was virtually integrated into the German one—and contractual obligations ensured a continuous flow of Austrian electricity to Germany until 1949.^22^ Allied forces in Germany willingly used this electricity, but were challenged by Switzerland and France, who wanted a share as well. Their plea was turned down, and the old contract remained in use.^23^ Disagreements also arose over how to exploit the Ruhr, with various schemes circulating ranging from internationalizing the Rhineland to completely dismantling the Ruhr industry (Bührer 1986). US Treasure Secretary Henry Morgenthau, Jr. advocated the latter in order to strip Germany of its industrial strength, which he thought to be epitomized by the Rhineland. This corresponded with French ideas, whose Rhineland policy was dominated by security interests and access to coal (Milward 1984). The Morgenthau plan was checked by the influential Military Governor of the American zone in Germany, General Lucius Clay, who opposed any international usage of Ruhr coal resources.^24^ By way of compromise, the Rhineland was divided between the French occupational zone (the eastern bank of the Rhine) and the British zone. The Ruhr area was placed under the International Ruhr Authority, to be replaced by the European Coal and Steel Community in 1952 which further arranged the international pooling of coal resources (Yoder 1955).

Another development that stimulated European cooperation in the field of electricity was the Marshall Plan. The US-sponsored aid program aimed to increase electricity production by nearly 70,000 million kWh by 1951. This was tried through the Organization for European Economic Cooperation (OEEC 1948), the institution in charge of distributing the Marshall Plan funds in Europe. The strategy was twofold; for one, the OEEC countries proposed a number of nationally-oriented electricity projects, but for another, urged by the US, the OEEC stimulated European cooperation through the so-called International Program. This Program consisted of nine power plants that utilized international resources (border rivers, streams of water from mountain ranges on borders), and were commonly financed and owned, to supply various countries (CEEC 1947).

Next to Marshall Plan funding, the International Program also caught the eye of Unternehmergeschäft-companies, who offered their services. Elektrobank, for example, contacted Marshall Plan officials in Washington, D.C. with a proposal to construct five plants on the High Rhine between Switzerland and Germany, supplying up to 1400 million kWh per year to both countries.^25^ The Belgian Unternehmersgeschäft SOFINA expressed its interest to invest in Austrian power plant projects to both the OEEC and United Nations.^26^

The International Program never came to fruition though. The fate of one such international power plant, Fessenheim on the French bank of the Upper Rhine, is illustrative for the International Program as a whole. Eventually built in 1956, the plant was a national project with French Marshall funds, together with two Rhine plants originally foreseen in the inter-war French Grand Canal d’Alsace plan: Ottmarsheim (1952) and Vogelgrün (1959) (Cioc 2002). Although the US regarded the International Program as the most important element of its electricity program, it did not materialize mainly because of persistent economic nationalism. OEEC countries opposed international ownership structures and refuted the international exploitation of resources located within their territory, prioritizing their national projects instead. The national representatives on the Electricity Committee of the OEEC concluded that implementing the International Program was very difficult because of “political and diplomatically uncertainty”.^27^

Other forms of international cooperation did come into being, however. Whereas the French initially unilaterally pursued their Grand Canal plan again, these schemes were buried in 1956 after top-level negotiations between Adenauer and Mollet. This also heralded a new phase in bilateral cooperation, as four more plants were built thereafter on the Canal: Marckolsheim (1961), Rheinau (1963), Gerstheim (1967), and Strasbourg (1970) (Disco 2006; Moser 2004; Tümmers 1999). This essentially completed the initial French scheme.

For electricity, shortly after the war, various actors argued for more cross-border coordination of electricity production in Western Europe. A 1947 report concluded that cooperation between neighboring countries around the Rhine was “not technically satisfactory”—a conclusion supported by US observers.^28^ A Marshall Plan-sponsored technical aid mission proved to be instrumental to improve this situation. This mission brought twenty-five electrical engineers from Western Europe to the United States, in order to study dispatch centers, control rooms, and power plants in 1949 on the other side of the Atlantic. All participants worked as load dispatchers in their respective countries, and the mission’s chairman was the director of the Laufenburg power plant, Swiss engineer René Hochreutiner—again underlining the prominence of the Rhine power plants.^29^ The mission’s final report advised to expand the number of international connections, as well as increased international coordination. Subsequent discussions within the OEEC led to a consensus: western European electricity resources should be pooled, but while maintaining the regional and national structures of individual utilities.^30^

This consensus eventually materialized in the Union for the Coordination of the Production and Transport of Electricity (UCPTE) in May 1951. The UCPTE consisted of representatives of nationalized and public utilities from eight countries (Belgium, Germany, France, Italy, Luxembourg, the Netherlands, Austria and Switzerland), all authorized by their respective governments to negotiate cross-border flows of electricity. Crucially, the UCPTE membership was on the basis of personal representation by network operators and engineers from participating utilities, and not on nation-state representation. A main objective of the UCPTE was balancing seasonal shortages and surpluses between countries, as well as preventing the spreading of incidents across borders. The UCPTE also took the lead in enhancing communication between network operators and coordinating power plant maintenance among members.

During the first years, international cooperation expanded by using existing transmission lines as much as possible. This changed significantly at the end of the 1950s, as the electricity systems of UCPTE members started to operate under similar technical conditions (synchronous operation at a unified frequency of 50 Hz). This required new HV-lines and other equipment. Synchronous operation implies that the national (and regional) networks now operated as one single technological system, leading to high degree of interdependence between the national systems. Temporary shortages or surplus in one country triggered an automatic response in adjacent countries, and made the system more robust. This led to a very reliable electricity supply system, with no large cross-border disturbances until 2003 (van der Vleuten and Lagendijk 2010).

Interestingly enough, the power plants and transmission along the Rhine region formed the core of this new technological interdependency. In 1958 the so-called “star of Laufenburg” became operational; with this spot on the Rhine as it center, transmission lines branched out to connect the networks of France (Électricité de France, EDF), Western Germany (RWE), and several Swiss companies in a star-shaped manner (UCPTE 1977). The Laufenburg “star” reaffirmed the Rhine’s central position in the post-war system, which provided a stable basis for the UCPTE system.

The “star” also persisted amidst the profound changes over the last 25 years. One major transformation was set in motion by the European Commission (EC) with the 1985 Single European Act, leading to a liberalized electricity market within the Community. As a consequence, monopoly control and ownership of exchanges, power plants and transmission networks were abolished. In addition, the national systems—which were previously closed for competition—opened up to other electricity producers and sellers (Lagendijk 2011; Padgett 1992). As liberalization gradually abolished state-owned and state-controlled electricity monopolies in Europe, electricity companies with their base in the Rhineland—like RWE and E.ON—came out particularly strong. Not only do they now possess considerable assets outside their home country, they are able to hold positions among the top-ranking energy companies in the world—competing with oil and gas conglomerates.^31^

A second remarkable development was the enormous growth of the interconnected system. While in 1958 the Laufenburg “star” connected only three countries, by 1996 sixteen more countries were part of this system. The points of the “star” first extended towards southern Europe, to include the Iberian peninsula together with Yugoslavia and Greece in 1987. A second wave occurred after 1989, leading to the interconnection of former Socialist countries in Central and Eastern Europe. Radiating from this node on the Rhine, the UCPTE system now encompassed the European continent from Poland to Portugal, in a way similar to the Interwar proposals.

## Conclusions

Today the Rhine region continues to play a crucial role even within this extensive continental system, though in a different manner than at the beginning of the twentieth century. Electricity companies from that region, like RWE, now own power plants and transmission lines in various countries. They used the region’s resources as a stepping stone towards becoming European players. Key parts of the current-day European grid are also based on the Rhine’s resources. Electricity systems grew as industries matured along the Rhine, as the exploitation of water and coal resources intertwined with the development of the metallurgic and chemical sectors. Although its resources became heavily contested, first during a nationalist phase and again after 1945, the Rhine region’s networks paved the way towards a European-wide electricity system. The somewhat utopian schemes of the Interwar period first suggested that notion. Paradoxically perhaps, at the high of the nationalist phase, the Nazi energy demand helped to connect the Rhineland—Germany’s industrial and electricity power base—to adjacent countries and resources. After the war, the process of European interconnection was left to the newly-found UCPTE and the again the Rhine acted as an important axis in balancing shortages and surpluses within western Europe. Furthermore, since 1958 the Laufenburg “star” turned this location on the High Rhine into a central node in the expanding European system.

While retracing the Rhine’s role in the history of the European system, this article clearly shows that in order to be able to fully assess these non-national processes, one has to focus on non-state—transnational—actors as well. Such actors, like companies, engineers, and international organizations, were particularly influential during the twentieth century. Electricity companies and firms from heavy industry dominated the first decades of electrification, and set up the framework for (geographical) expansion in later years. Non-state actors like engineers, entrepreneurs, and financiers operated rather unhampered by national governments. Starting out by catering to local needs, the geographical scale of cooperation increased due to innovative financial-engineering structures, new high voltage technologies, and bigger power plants. Companies took the lead in utilizing the river’s riches, sub-national states provided the legal grounds, and internationally-minded engineers provided the expertise to undertake the actual work. The UCPTE was very influential in constructing a technical interconnection between western European countries, and operated as a non-state actor composed of engineers. The 1958 Laufenburg “star” reaffirmed the Rhine’s central position, making it the backbone for the UCPTE’s system. In today’s European liberalized energy market, electricity companies again appear in charge, only restrained by national and European regulatory bodies.

But dismissing the nation-state’s role entirely would be throwing away the baby with the bathwater. During the Interwar years—the nationalist phase—the power of the nation-state was felt quite strongly, despite the brief surge of European plans. National authorities took the initiative in steering the electricity system to benefit the national economy, and issued protectionist laws. The Rhine, too, became the object of French nationalist plans on the Grand Canal d’Alsace. During post-war reconstruction, when electricity was perceived to be a key sector, national authorities successfully obstructed the Marshall Plan’s International Program. Even with international pooling of resources taking place, with the UCPTE being de facto responsible for international electricity flows, national energy policies were left largely intact. One should thus see the international framework as supplementing and boosting the national electricity systems.

This should thus be regarded the main advantage of using a transnational approach. It enables one to rethink the role of the nation-state during certain phases, and at the same time exposes developments that went unnoticed thus far. National histories of electrification have not only largely missed out on the gradual construction of a European system, they also failed to notice the significance of the Rhine region. While to electrical engineers the Laufenburg “star” shone bright, the ongoing process of European system-building went unnoticed by European citizens—and historians, I must add. Hence the term “hidden integration” seems apt to describe the development of a European electricity system in the twentieth century, and the Rhine’s role therein. The central role of the anonymous control rooms at Brauweiler and Laufenburg testify to that.

## Footnotes

1 For the nationalizing tendencies of natural resources, also see White (1999).

2 To name but a few references, see Clavin (2005), Iriye (2013), Patel (2004), Saunier (2008), and Van der Vleuten (2008). My ideas on this matter have been shaped and sharpened by ongoing discussions with Patricia Clavin, Kiran Klaus Patel, Pierre-Yves Saunier, Erik van der Vleuten and Johan Schot.

3 See as an important starting point, Tempelhoff et al. (2009). Also see Coopey and Tvedt (2006) and Tvedt and Jakobsson (2006).

4 *A History of Water*, published by I.B. Tauris, with Terje Tvedt as series editor. See the book series website, http://waterhistory.b.uib.no/.

5 Another recent and key title is Zeisler-Vralsted (2014).

6 The literature on the United States is particularly extensive. See for example Billington and Jackson (2006), McCool (2012), Pisani (2002), Reisner (1986), White (1995) and Worster (1985). For European countries see amongst others, Alexandre Giandou, *La Compagnie nationale du Rhône: 1933-1998. Histoire d’un partenaire régional de l'état* (Grenoble: Presses universitaires de Grenoble, 1999); Erik Swyngedouw, “‘Not A Drop of Water…’: State, Modernity and the Production of Nature in Spain, 1898-2010”, *Environment and History* 20, no. 1 (February 1, 2014): 67–92; David Blackbourn, *The Conquest of Nature: Water, Landscape, and the Making of Modern Germany* (New York: Norton, 2006); Erik van der Vleuten and Cornelis Disco, “Water Wizards: Reshaping Wet Nature and Society”, *History and Technology* 20, no. 3 (September 2004): 291–309.

7 “Qui veut écrire sur le Rhin, en historien, doit d’abord exorciser des fantômes […]”.

8 Also see the work by Nil Disco (2013) and Cornelis Disco (2008, 2010).

9 For a recent study of a river with “changing” nationality, see Korjonen-Kuusipuro (2011).

10 See for example White (1999) Another case, related to riverine uses for electricity production, is Switzerland and documented by David Gugerli in his (1996).

11 One notable exception is the history of electrical engineering communities, that have been international from very early on. For example see Barjot and Kurgan (2004), and Bouneau (2004).

12 According to Clavin, transnationalism is not only about international organizations, but also about people and “the social space that they inhabit, the networks they form and the ideas they exchange”. See Clavin (2005).

13 Also see the recent book by Schot and Kaiser (2014) that builds on this very same notion.

14 Only the latter materialized, via the Rhine-Main-Danube canal, completed in 1972. See Disco (2010).

15 This was not the first power plant on the Rhine, however. Already as early as 1850-51, Swiss watchmaker, inventor, and entrepreneur Heinrich Moser built the first power plant on the High Rhine at Schaffhausen. The plant had a limited capacity of 80 horse power, and supplied local industry. In the 1860 s this was extended to a dam equipped with more powerful generators (Paquier 2009).

16 Legge (1931) explains that 60 kV could transmit electricity over a distance of 100 km with a loss of approximately 10 percent, which was seen as the upper limit. 110 and 220 kV made it economically interesting to connect plants and consumers up to 200 to 400 km.

17 Storage dams are able to hold water and release it at particular times.

18 Treaty of Versailles, 2 June 1918, Article 358b.

19 Elsewhere Johan Schot and myself have labeled this as part of Technocratic Internationalism. See Schot and Lagendijk (2008).

20 Austrian State Archives, Vienna, Collection Wasserwirtschaftsstelle für das untere Donau-gebiet, 1940-1942, Box 134, ‘Niederschrift über die Sitzung des Fachausschusses I (Wasserkraftplannung) am 27 and 28.1.1942 in Berlin, Pariser Platz 5a’.

21 Schweizerisches Bundesarchiv, Bern, Switzerland, 8E 8190, Amt für Energiewirtschaft 1930–1969, File 83: Ausfuhrbewilligungen (81, 81.1, 82), Folder: Allgemeines 1930–1951, 'Report of a Meeting held on 22 June 1945 in Laufenburg'.

22 The National Archives, Kew (hereafter: NA), Foreign Office File 371/64976, 1947, Austria, File No. 2425, Memorandum by J.W. Nicholls of German Trade Department, 20 August 1947.

23 National Archives of the United States, College Park, Maryland (hereafter NARA), Record Group (RG) 469: Records of U.S. Foreign Assistance Agencies, File 2.2: Subject files and Issuances of the Organization and Management Division, Box 14, Cable from Vienna to ECA Administrator, 6 July 1949.

24 NARA, RG 469, file 2.2, box 1, DeForest to Perkins, 13 November 1948.

25 NARA, RG 469, Industry Division (1948–1951), Entry nr. 986, Box 1, Confidential Memo DeForest to Cisler, 26 November 1948.

26 SOFINA was primarily interested in Alpine region. Archive of the United Nations Organization Geneva, Switzerland (hereafter UNOG), GX: Economics, 19/6/1/4 – 3815, Electric Power: Transmission, Operation and Interconnexions, Exchanges of Electric Power, Box 50, Jacket 1, Document W/EP.6/7, ‘Report of an informal meeting held at Innsbrück on July 6th, 1949, to discuss private capital participation in Austrian Power Projects’, 13 July 1949.

27 Historical Archives of the European Union, Florence, Italy (hereafter HAEU), fonds OEEC, file 1157.3, document EL/M(48), ‘Minutes of the 10^th^ meeting’, 10 September 1948.

28 NA, Foreign Office 371/57960, 1946 – Economic, File No. 1879, Folder: UR 6870/1879/53, Public Utilities Panel, Draft Minutes of the Fifteenth Meeting (EEC(47) 81), 14 July 1947, 18. For the American perspective, see NARA, RG 469, file 2.2, box 1, Cisler and DeForest to Perkins, 26 October 1948.

29 A reflection of the mission is given in (OEEC 1950).

30 HAEU, fonds OEEC, file 1156.1, document EL(50)16, ‘Council recommendation concerning the working of the international power pool’, 26 March 1950; and *Ibidem.*, document EL(50)22, ‘Draft agreement between the countries of Western Europe for the joint operation of electric power resources’, 20 May 1950.

31 The two German companies E.ON (the outcome of a 2000 merger between the companies VIAG and VEBA) and RWE are respectively numbers 26 and 49 on the PFC Energy Top 50. See PFC Energy (2013).
